# Synthesis of Phthalimide Derivatives as Potential PPAR-γ Ligands

**DOI:** 10.3390/md14060112

**Published:** 2016-06-08

**Authors:** So Hyeon Eom, Sen Liu, Mingzhi Su, Tae Hwan Noh, Jongki Hong, Nam Deuk Kim, Hae Young Chung, Min Hye Yang, Jee H. Jung

**Affiliations:** 1 College of Pharmacy, Pusan National University, Busan 46241, Korea; god5425um@naver.com (S.H.E.); ls691392008@sina.com (S.L.); sumingzhi0310@gmail.com (M.S.); notea151@naver.com (T.H.N.); nadkim@pusan.ac.kr (N.D.K.); hyjung@pusan.ac.kr (H.Y.C.); 2 College of Pharmacy, Kyung Hee University, Seoul 02447, Korea; jhong@khu.ac.kr

**Keywords:** PPAR-γ agonist, paecilocin A, type 2 diabetes, phthalimide, adipogenesis, 3T3-L1

## Abstract

Paecilocin A, a phthalide derivative isolated from the jellyfish-derived fungus *Paecilomyces variotii*, activates PPAR-γ (Peroxisome proliferator-activated receptor gamma) in rat liver Ac2F cells. Based on a SAR (Structure-activity relationships) study and *in silico* analysis of paecilocin A-mimetic derivatives, additional *N*-substituted phthalimide derivatives were synthesized and evaluated for PPAR-γ agonistic activity in both murine liver Ac2F cells and in human liver HepG2 cells by luciferase assay, and for adipogenic activity in 3T3-L1 cells. Docking simulation indicated **PD6** was likely to bind most strongly to the ligand binding domain of PPAR-γ by establishing crucial H-bonds with key amino acid residues. However, in *in vitro* assays, **PD1** and **PD2** consistently displayed significant PPAR-γ activation in Ac2F and HepG2 cells, and adipogenic activity in 3T3-L1 preadipocytes.

## 1. Introduction

Type 2 diabetes mellitus (T2DM) is one of the most common serious metabolic diseases. T2DM results in insulin resistance and damages β-cell function [1]. Consequently, T2DM exhibits impaired insulin secretion, increased hepatic glucose production, and decreased insulin-stimulated glucose uptake by peripheral tissues [2]. Peroxisome proliferator-activated receptors (PPARs) are ligand-activated nuclear receptors that regulate transcription factors. The three known PPAR isoforms are PPAR-α, -β/δ, and -γ, and PPAR-γ is known to increase insulin sensitivity in adipose, muscle, and hepatic tissues [3,4,5]. Thiazolidinediones (TZDs) (e.g., troglitazone, pioglitazone, and rosiglitazone) are a class of PPAR-γ agonists that are used as anti-diabetic drugs [6] and improve insulin resistance in liver and skeletal muscle and preserve pancreatic β-cell function [7]. However, TZDs have been associated with undesirable side effects, such as hepatotoxicity and edema [8,9]. Therefore, research interest in this area is focused on the discovery of novel PPAR-γ agonists with fewer adverse effects [10].

**Figure 1:**
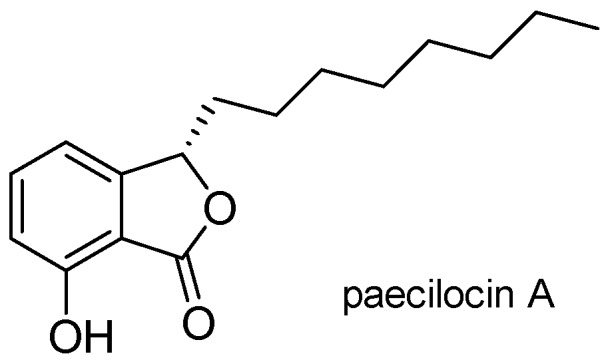
Chemical structure of paecilocin A.

In our previous study, paecilocin A (Figure 1), a new phthalide derivative isolated from the jellyfish-derived fungus *Paecilomyces variotii*, showed PPAR-γ agonistic activity [11]. Notably, the plant-derived phthalides 3-butylphthalide, 3-butylidenephthalide, and ligustilide have been patented as effective agents for the treatment of diabetes mellitus type 2 [12]. The mechanism responsible for their activities was surmised to be PPAR-γ activation. The findings of our previous pharmacophore study suggested the 3-hydroxy phthalide moiety of paecilocin A serves as a hydrophilic head that binds to the ligand binding domain (LBD) of PPAR-γ and forms H-bonds with key amino acid residues [13]. Based on analyses of the structures of paecilocin A and TZDs, *N*-substituted phthalimide derivatives (**PD1**–**PD4**) were synthesized and evaluated [14].

In the present study, additional *N*-substituted phthalimide derivatives **PD5** and **PD6** were synthesized based on *in silico* analysis. In a docking simulation, **PD5** and **PD6**, which are hydroxylated at C-4 rather than at C-3, showed high affinity and formed H-bonds with the key amino acid residues of the LBD of PPAR-γ. Therefore, **PD5** and **PD6** were synthesized as potential PPAR-γ agonists and compared with **PD1**–**PD4**. Since PPAR-γ is a key transcriptional factor for the inductions of adipogenic marker genes [15], these analogues were also evaluated for adipogenic activity on 3T3-L1 preadipocytes.

## 2. Results and Discussion

### 2.1. Docking Simulation

**Figure 2:**
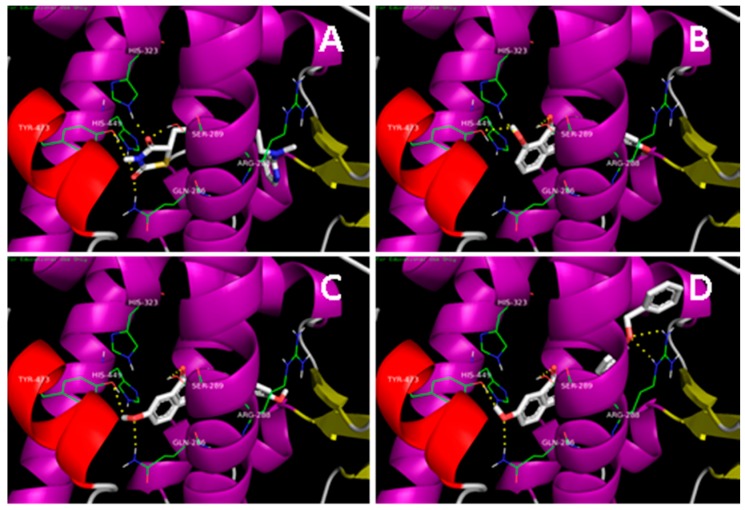
Docking simulations of rosiglitazone, PD1, PD5, and PD6 with the LBD of PPAR-γ. (**A**) Rosiglitazone interacts with key amino acid residues (Tyr^473^, His^449^, His^323^, Ser^289^, and Gln^286^) in the PPAR-γ binding pocket (−8.6 kcal/moL). The docking pose of rosiglitazone was simulated using the reported co-crystal structure (PDB: 2PRG); (**B**) **PD1** (−8.3 kcal/moL), interacts with key amino acid residues (Tyr^473^, His^323^, and Ser^289^) in the PPAR-γ binding pocket; (**C**) **PD5** (−8.1 kcal/moL) interacts with key amino acid residues (Tyr^473^, Gln^286^, and Ser^289^) in the PPAR-γ binding pocket; (**D**) **PD6** (−10.0 kcal/moL) interacts with key amino acid residues (Tyr^473^, Ser^289^, Gln^286^ and Arg^288^) in the PPAR-γ binding pocket.

In our previous study, a series of *N*-substituted phthalimide derivatives, including **PD1**–**PD4**, were synthesized based on the potential pharmacophore of paecilocin A, and **PD1** was proposed as an interesting lead [14]. Further docking analysis of derivatives (**PD5** and **PD6**) hydroxylated at C-4 showed that they both bind to LBD of PPAR-γ and form H-bonds with key amino acid residues (Figure 2). In particular, docking analysis showed **PD6** forms H-bonds with Tyr473, Ser289, and Gln286 in the PPAR-γ binding pocket with higher binding affinity than **PD1**. Therefore, **PD5** and **PD6** were synthesized as potential PPAR-γ agonists and compared with **PD1**–**PD4**.

### 2.2. Synthesis of Analogues

**Scheme 1:**
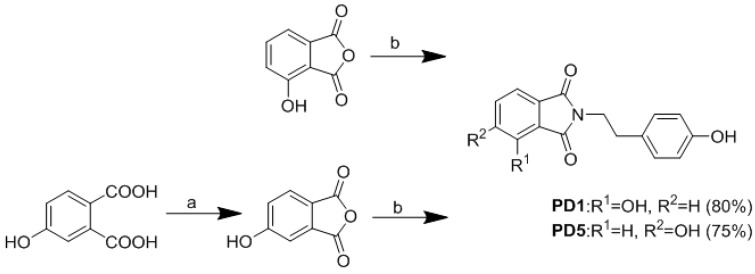
Synthesis of phthalimide derivatives. Reagents and conditions: (**a**) Ac_2_O, pyridine, rt, 12 h; (**b**) tyramine, aqueous CH_3_COOH, reflux 85 °C, overnight.

**Scheme 2:**
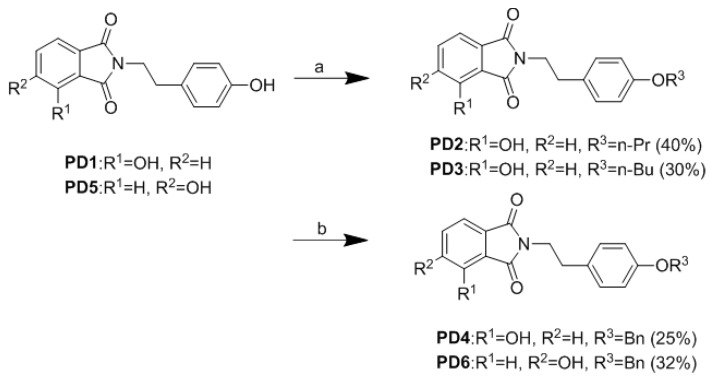
Synthesis of phthalimide derivatives. Reagents and conditions: (**a**) RI (*n*-PrI, *n*-BuI), Ag_2_O, CH_3_CN, 12h; (**b**) benzyl chloride, K_2_CO_3_, NaI, DMF, rt, 4 h.

*N*-substituted phthalimide can be prepared by heating anhydride with various *N*-containing reagents [16]. According to the previous study, the introduction of hydroxyl groups would allow the phthalimide moiety to be more effective as a polar head group and this modification could be further extended using linker and tail groups to generate potential PPAR-γ ligands. Compounds **PD1**–**PD4** were prepared according to the protocol in our previous report [14]. Then 3-Hydroxyphthalic anhydride was treated with tyramine in aqueous glacial acetic acid to afford phthalimide derivative **PD1**. Compound **PD1** was further derivatized by treating with iodopropane, iodobutane, or benzyl chloride to afford **PD2**, **PD3**, and **PD4**, respectively. Commercially available 4-hydroxyphthalic acid was added to acetic anhydride and then treated with pyridine to afford 4-hydroxyphthalic anhydride. Then 4-Hydroxyphthalic anhydride was subsequently treated with tyramine in aqueous glacial acetic acid to afford **PD5** (Scheme 1 and Scheme 2).

Compound **PD6** was synthesized by treating **PD5** with benzyl chloride at room temperature for 4 h (Scheme 2).

### 2.3. Biological Evaluation

#### 2.3.1. PPAR-γ Activation

**Figure 3:**
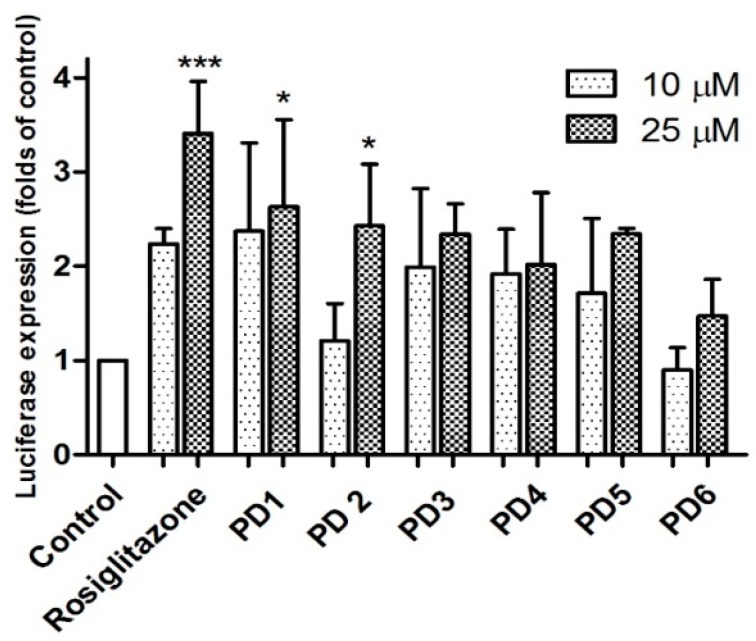
PPAR-γ activation by PD1–PD6 in Ac2F cells. *In vitro* assay of PPAR-γ activation by phthalimides **PD1**~**PD6** and rosiglitazone at concentrations of 10 and 25 μM in rat liver Ac2F cells. Cells were transiently transfected by PPRE together with pFlag-PPAR-γ1. Rosiglitazone was used as positive control to monitor the activation of luciferase reporter. Control cells were transfected with plasmid containing PPRE and pcDNA3. Luciferase expressions (folds of control) are presented as means ± SDs (*n* = 3). * *p* < 0.05 *versus* control. *** *p* < 0.001 *versus* control.

PPARs are ligand-activated transcription factors. PPAR-γ binding of ligands results in up-regulation of the expression levels of mRNAs encoded by PPAR target genes [17]. In the luciferase assay, activation of a receptor by ligands leads to the induction of reporter enzyme expression, which can be assayed using a photometric plate reader. Synthesized **PD5** and **PD6** were subsequently evaluated for PPAR-γ activation using a luciferase assay in rat liver Ac2F cells. Initially, they were compared with rosiglitazone and **PD1**–**PD4** with respect to PPAR-γ activation at concentrations of 10 and 25 μM (Figure 3). Compound **PD5** induced significantly more PPAR-γ activation than untreated controls, but it was not as potent as **PD1**. Furthermore, **PD6** was not as potent as was indicated by docking simulation. It would appear that the hydroxyl at C-4 of the phthalimide moiety is not as useful for binding to the LBD of PPAR-γ in Ac2F cells as hydroxyl at C-3.

**Figure 4:**
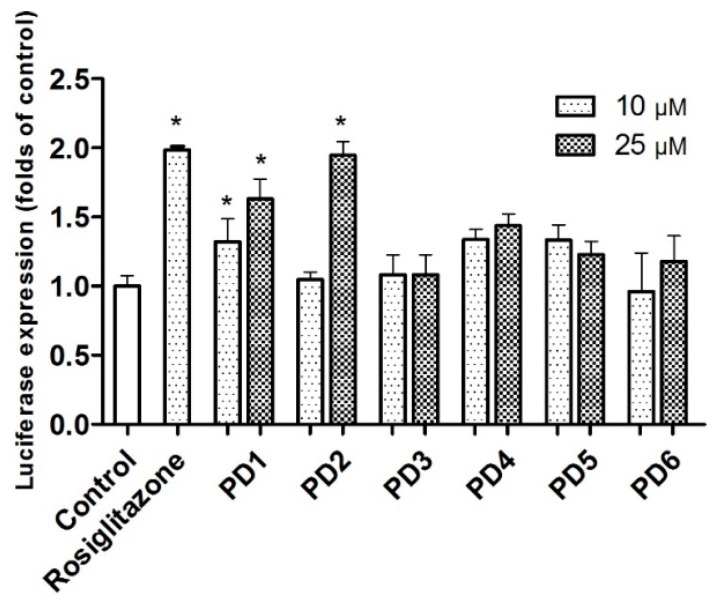
PPAR-γ activation by PD1–PD6 in HepG2 cells. *In vitro* PPAR-γ activations by phthalimides **PD1**–**PD6** and rosiglitazone were investigated at 10 μM and 25 μM in HepG2 cells. Cells were transiently transfected with PPRE plus pFlag-PPAR-γ1. Rosiglitazone was used as positive control to monitor the activation of luciferase reporter. Luciferase expressions (folds of untreated controls) are presented as means ± SDs (*n* = 3). * *p* < 0.05 *versus* untreated controls.

Analogues **PD1**–**PD6** were further evaluated for PPAR-γ activation using human liver HepG2 cells at concentrations of 10 and 25 μM (Figure 4). **PD1** and **PD2** induced significant PPAR-γ activation at 25 μM. Although **PD1**~**PD6** were not as potent as rosiglitazone in HepG2 or Ac2F cells, **PD1** consistently significantly activated PPAR-γ in both cell lines.

#### 2.3.2. Effect on Adipocyte Differentiation in 3T3-L1 Cells

**Figure 5:**
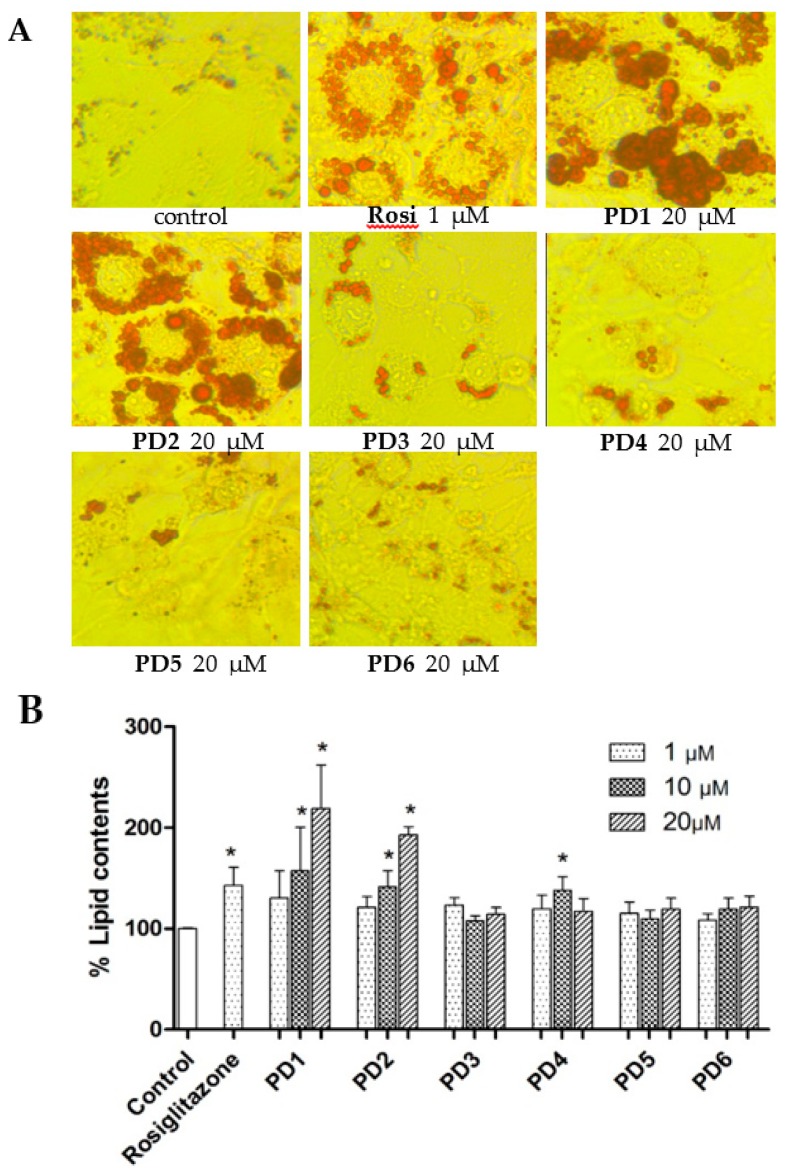
Effects of PD1–PD6 on adipocyte differentiation of 3T3-L1 cells. The 3T3-L1 cells were exposed to 10% FBS/DMEM containing 10 μg/mL insulin and 1 μM dexamethasone in the presence of various concentrations (1, 10, or 20 μΜ) of **PD1**–**PD6** or rosiglitazone (1 μΜ). After eight days, treated cells were stained with Oil red O for morphological assessment by microscopy (**A**) and lipid contents was determined using an iMark Microplate Absorbance Reader (**B**). The results shown are representative of three independent experiments, and are presented as means ± SDs (*n* = 3). * *p* < 0.05 *versus* untreated controls.

PPAR-γ is a key transcription factor for the induction of adipogenic marker genes, and PPAR-γ agonists induce adipogenesis of preadipocytes into mature adipocytes. To examine whether **PD1**–**PD6** induce adipocyte differentiation, 3T3-L1 preadipocytes were treated with insulin and dexamethasone in the presence of various concentrations of **PD1**–**PD6** for eight days. Rosiglitazone was used as a positive control, as it is known to have a marked adipogenic effect on preadipocytes *in vitro*. Significant induction of adipocyte differentiation by rosiglitazone was observed at a concentration of 1 µM by Oil red O staining (Figure 5A), and this correlated well with observed increases in lipid accumulation (Figure 5B). **PD1** and **PD2** significantly stimulated adipogenesis in a concentration-dependent manner (1~20 μM), whereas **PD3**–**PD6** did not significantly increase adipogenesis. Furthermore, the adipogenic profiles of **PD1**–**PD6** correlated fairly well with PPAR-γ activations in Ac2F and HepG2 cells.

## 3. Experimental Section

### 3.1. Chemistry

The ^1^H and ^13^C NMR spectra were recorded on a Varian Unity 400 MHz NMR spectrometer, and chemical shifts are reported with respect to residual solvent or deuterated solvent peaks (*δ_H_* 3.30 and *δ_C_* 49.0 for CD_3_OD, *δ_H_* 7.24 and *δ_C_* 76.8 for CDCl_3_). ESI MS data were obtained using a Agilent 6530 accurate-mass Q-TOF MS spectrometer .HPLC was performed using a YMC ODS-H80 column (250 × 10 mm, 4 μm, 80 Å) or a C18-5E Shodex packed column (250 × 10 mm, 5 μm, 100 Å) and a Shodex RI-71 detector. All reagents were purchased from Sigma-Aldrich and used as received.

A mixture of tyramine (1.2 equivalents) and 3-hydroxypthalic anhydride in aqueous glacial acetic acid (1 M) was stirred under reflux overnight. Products were precipitated by adding water, filtering, and washing thoroughly with water. Residues were diluted with MeOH, dried using MgSO_4_, and evaporated to provide the crude products [12].

3-Hydroxy-*N*-(*p*-hydroxy-phenethyl)phthalimide (**PD1**). White powder; ^1^H NMR (400 MHz, CD_3_OD): *δ* 2.82 (t, *J* = 7.2 Hz, 2H), 3.75 (t, *J* = 7.6 Hz, 2H), 6.64 (d, *J* = 8.4 Hz, 2H), 6.98 (d, *J* = 8.4 Hz, 2H), 7.08 (d, *J* = 8.0 Hz, 1H), 7.24 (d, *J* = 7.2 Hz, 1H), *δ* 7.53 (dd, *J* = 7.2, 7.6 Hz, 1H). ESI MS *m*/*z* 284.0912 [M + H]^+^.

To a solution of 3-hydroxy-*N*-(*p*-hydroxy-phenethyl)phthalimide **PD1** (13 mg, 0.046 mM) in CH_3_CN (1.5 mL), RI (CH_3_(CH_2_)_2_I: 9 μL, *ca.* 0.09 mM; CH_3_(CH_2_)_3_I: 10 μL, *ca.* 0.09 mM) and Ag_2_O (10 mg, 0.04 mM) were added. The mixture was then heated under reflux with stirring for 12 h. Solid material was removed by filtration and solvent by evaporation. The solid material so obtained was purified by RP-HPLC using 90% aqueous MeOH as eluent to give **PD2** and **PD3**.

3-Hydroxy-*N*-(*p*-propoxy-phenethyl)phthalimide (**PD2**). White powder; ^1^H NMR (400 MHz, CDCl_3_): *δ* 1.09 (t, *J* = 7.2 Hz, 3H), *δ* 1.92 (m, 2H), *δ* 2.89 (m, 2H), *δ* 3.82 (t, *J* = 6.0 Hz, 2H), *δ* 4.13 (t, *J* = 6.0 Hz, 2H), *δ* 6.74 (d, *J* = 6.4 Hz, 2H), *δ* 7.11 (d, *J* = 6.8 Hz, 2H), *δ* 7.16 (d, *J* = 8.0 Hz, 1H), *δ* 7.39 (d, *J* = 7.2 Hz, 1H), *δ* 7.61 (t, *J* = 7.8 Hz, 1H); ^13^C NMR (100 MHz, CDCl_3_): *δ* 168.2, 167.1, 156.4, 154.4, 136.0, 134.4, 130.4, 130.2, (2C overlapped), 118.7, 117.5, 115.5, (2C overlapped), 115.4, 70.9, 39.5, 33.9, 22.5, 10.5. ESI MS *m*/*z* 326.1381 [M + H]^+^.

3-Hydroxy-*N*-(*p*-butoxy-phenethyl)phthalimide (**PD3**). White powder; 1H NMR (400 MHz, CDCl_3_): *δ* 0.99 (t, *J* = 7.4 Hz, 3H), *δ* 1.52 (m, 2H), *δ* 1.87 (m, 2H), *δ* 2.89 (m, 2H), *δ* 3.82 (m, 2H), *δ* 4.17 (t, *J* = 6.8 Hz, 2H), *δ* 6.75 (d, *J* = 8.0 Hz, 2H), *δ* 7.12 (d, *J* = 8.2 Hz, 2H), *δ* 7.17 (d, *J* = 8.6 Hz, 1H), *δ* 7.39 (d, *J* = 7.6 Hz, 1H), *δ* 7.61 (m, 1H); ^13^C NMR (100 MHz, CDCl_3_): *δ* 168.2, 167.1, 156.4, 154.4, 136.0, 134.4, 130.4, 130.2, (2C overlapped), 118.6, 117.5, 115.5, (2C overlapped), 115.4, 69.2, 39.5, 33.9, 31.1, 19.3, 14.0. ESI MS *m*/*z* 340.1543 [M + H]^+^.

To a suspension of 3-hydroxy-*N*-(*p*-hydroxy-phenethyl) phthalimide **PD1** (13.4 mg, 0.047 mM), K_2_CO_3_ (6.5 mg, 0.047 mM), and NaI (2.6 mg, 0.02 mM) in DMF (2 mL) was added benzyl chloride (6.0 μL, *ca.* 0.06 mM). The mixture was then stirred for 30 min at 0 °C and for 4 h at room temperature, acidified with aqueous 6 M HCl, and extracted with EtOAc. The organic layer was sequentially washed with H_2_O and brine, dried with MgSO_4_, and evaporated to give the crude product, which was purified by RP-HPLC using 85% aqueous MeOH as eluent to give **PD4**.

3-Hydroxy-*N*-(*p*-benzyl-phenethyl)phthalimide (**PD4**). White powder; ^1^H NMR (400 MHz, CDCl_3_): *δ* 2.91 (t, *J* = 8.0 Hz, 2H), *δ* 3.85 (t, *J* = 7.2 Hz, 2H), *δ* 5.33 (s, 2H), *δ* 6.75 (d, *J* = 8.0 Hz, 2H), *δ* 7.12 (d, *J* = 8.4 Hz, 2H), *δ* 7.19 (d. *J* = 8.4 Hz, 1H), *δ* 7.33 (m, 1H), *δ* 7.40 (t, *J* = 7.2 Hz, 3H), *δ* 7.49 (d, *J* = 7.6 Hz, 2H), *δ* 7.58 (t, *J* = 8.4 Hz, 1H); ^13^C NMR (100 MHz, CDCl_3_): *δ* 168.0, 166.9, 155.7, 154.4, 136.0, 136.0, 134.4, 130.4, 130.2, (2C overlapped), 128.9, (2C overlapped), 128.3, 127.0, (2C overlapped), 119.5, 118.1, 115.9, 115.5, (2C overlapped), 71.0, 39.5, 33.9 . ESI MS *m*/*z* 374.1387 [M + H]^+^.

Next 4-Hydroxy phthalic acid (182 mg, 1.0 mM) was added to acetic anhydride (5 mL) in a flask, and then 1 mL of pyridine was added with stirring in an iced-water bath. After 10 min, the reaction mixture was stirred for 12 h at room temperature, and the resultant solution was slowly acidified with 0.01 M HCl with stirring in an iced-water bath. The large amount of precipitate produced was collected by filtration [18]. A mixture of tyramine (164 mg, 1.2 mM) and this precipitate in aqueous glacial acetic acid (1 M) was then stirred under reflux overnight. Solvent was removed by evaporation, and the solid material so obtained was purified by RP-HPLC using 65% aqueous MeOH as eluent to give **PD5**.

4-Hydroxy-*N*-(*p*-hydroxy-phenethyl)phthalimide (**PD5**). White powder; ^1^H NMR (400 MHz, CD_3_OD): *δ* 2.83 (m, 2H), *δ* 3.78 (m, 2H), *δ* 6.66 (d, *J* = 8.8 Hz, 2H), *δ* 7.00 (d, *J* = 8.4 Hz, 2H), *δ* 7.07 (dd, *J* = 8.2, 2.2 Hz, 1H), *δ* 7.14 (d, *J* = 2.0 Hz, 1H), *δ* 7.64 (d, *J* = 8.0 Hz, 1H); ^13^C NMR (100 MHz, CDCl_3_): *δ* 169.7, 169.6, 164.9, 157.1, 136.0, 130.8, (2C overlapped), 130.3, 126.1, 123.6, 121.3, 116.2, (2C overlapped), 110.9, 40.5, 34.6. ESI MS *m*/*z* 284.0913 [M + H]^+^.

To a suspension of 4-hydroxy-*N*-(*p*-hydroxy-phenethyl) phthalimide **PD5** (30.2 mg, 0.106 mM), K_2_CO_3_ (14.7 mg, 0.106 mM), and NaI (6.89 mg, 0.053 mM) in DMF (5 mL) was added benzyl chloride (13.5 μL, *ca.* 0.13 mM). The mixture was then stirred for 30 min at 0 °C and for 4 h at room temperature, acidified with aqueous 6 M HCl, and extracted with EtOAc. The organic layer was sequentially washed with H_2_O and brine, dried with MgSO_4_ and evaporated to give the crude product, which was purified by RP-HPLC using 85% aqueous MeOH as eluent to give **PD6**.

4-Hydroxy-*N*-(*p*-benzyl-phenethyl)phthalimide (**PD6**). White powder; ^1^H NMR (400 MHz, DMSO-*d*6): *δ* 2.77 (t, *J* = 7.2 Hz, 2H), *δ* 3.70 (t, *J* = 7.2 Hz, 2H), *δ* 5.29 (s, 2H), *δ* 6.62 (d, *J* = 8.4 Hz, 2H), *δ* 6.95 (d, *J* = 8.4 Hz, 2H), *δ* 7.35 (m, 2H), *δ* 7.41 (m, 3H), *δ* 7.48 (m, 2H), *δ* 7.76 (d, *J* = 8.4 Hz, 1H), *δ* 9.21 (s, 1H); ^13^C NMR (100 MHz, DMSO-*d*6): *δ* 167.4, 167.3, 163.4, 155.8, 136.1, 134.1, 129.5, (2C overlapped), 128.6, (2C overlapped), 128.3, 128.2, 127.9, (2C overlapped), 124.9, 123.5, 120.5, 115.2, (2C overlapped), 108.9, 70.2, 39.8, 32.9. ESI MS *m*/*z* 384.1385 [M + H]^+^.

### 3.2. Cell Culture

Rat liver (Ac2F) cells were obtained from the American Type Culture Collection (ATCC, Rockville, MD, USA) were cultured in Dulbecco’s Modified Eagle Medium (DMEM, Nissui, Tokyo, Japan) containing 2 mM l-glutamine, 100 μg/mL streptomycin (HyClone, Logan, UT, USA), 2.5 mg/L amphotericin B (HyClone, Logan, UT, USA), and 10% heat-inactivated fetal bovine serum (FBS, Gibco, Grand island, NE, USA) at 37 °C in a humidified 5% CO_2_. Human hepatoma (HepG2) cells were from the ATCC and maintained in DMEM supplemented with 10% FBS, 100 units/mL penicillin, and 100 μg/mL streptomycin at 37 °C with 5% CO_2_. Preadipocytes (3T3-L1) was purchased from ATCC CL-173 and was grown and maintained in DMEM containing 10% Bovine calf serum (BCS, Gibco,), 100 units/mL penicillin, and 100 μg/mL streptomycin at 37 °C in a humidified 5% CO_2_.

### 3.3. Luciferase Assay

TK-PPRE × 3-luciferase reporter plasmid containing three copies of PPAR response element (PPRE) in acyl CoA oxidase promoter was generously donated by Dr. Christopher K. Glass (University of California, San Diego, CA, USA). pcDNA3 expression vector and full-length human PPAR-γ1 expression vector (pFlag-PPAR-γ1) were generously donated by Dr. Chatterjee (University of Cambridge, Addenbrooke’s Hospital, Cambridge, UK). For luciferase assays, cells at 70%–80% confluence were transfected in a 48-well plate (5 × 10^4^ cells/well) with effector plasmids and TK-PPRE × 3-luciferase reporter plasmid (1 μg/well) plus pcDNA3 (0.1 μg/well) or pFlag- PPAR-γ1 (0.1 μg/well) using Lipofectamine™ 2000 (Invitrogen Co., Carlsbad, CA, USA), according to the manufacturer’s instructions. After transfection for 4 h, conditioned media were replaced with complete medium, and cells were incubated for an additional 20 h. Media were then removed, and cells were treated with rosiglitazone or test compounds in serum-free media for 6 h. Cells were then lysed and assayed using the ONE-Glo™ Luciferase Assay System (Promega, Madison, WI, USA). Luciferase activities were measured using a GloMax^®^-Multi Microplate Multimode Reader (Promega Co., Sunny Vale, CA, USA).

### 3.4. Molecular Docking Study

Docking calculations were performed using AutoDock Vina 1.1.2 software (The Scripps Research Institute, La Jolla, CA, USA). Default settings and the Vina scoring function were applied. For PPAR γ ligand preparation, Chem3D Ultra 8.0 software (Cambridge Soft Corporation, Cambridge, MA, USA) was used to convert the 2D structures of candidates into 3D structural data. Protein coordinates were downloaded from the Protein Data Bank (accession code: 2PRG). Chain A was prepared for docking using the Chimera 1.5.3 molecular modeling software package (National Institute of Health, Bethesda, MD, USA) by removing chain B, all ligands and water molecules (except water molecules 308, 399, 444, and 467), and calculating protein protonation states. Polar hydrogen and setting grid box parameters were added using MGLTools 1.5.4 (The Scripps Research Institute, La Jolla, CA, USA). The analysis and visual investigation of ligand-protein interactions of docking poses were performed using PyMol v1.5 (Schrodinger LLC, New York, NY, USA).

### 3.5. Adipocyte Differentiation Assay

Preadipocytes (3T3-L1) were seeded in 48 well plates at a density of 5 × 10^4^ cells/well and maintained until two days post-confluence. After two days of confluency, medium was replaced with 10% FBS/DMEM containing 10 μg/mL insulin, 1 μΜ dexamethasone, and test samples at various concentrations (day 2). After two days, the medium was replaced with 10% FBS/DMEM containing 10 μg/mL insulin and test samples (1, 10, and 20 μΜ) (day 4). The culture was then maintained in 10% FBS/DMEM for an additional four days.

### 3.6. Quantification of Lipid Contents in 3T3-L1 Cells

After eight days of differentiation in the presence of samples, medium was removed, cells were carefully rinsed with PBS, fixed with 70% ethanol for 30 min, stained with Oil red O solution for 1 h, and washed with dH_2_O. Pictures were taken using a Optinity (Gyeonggi-Do, Korea) microscope. Oil red O was eluted by adding 200 μL of isopropanol on a rotary shaker for 5 min. Eluents were transferred to a 96-well plate and ODs were measured using a iMark Microplate Absorbance Reader (Bio-Rad Laboratories, Hercules, CA, USA) at 544 nm.

## 4. Conclusions

Several PPAR-γ agonists, such as troglitazone, pioglitazone, and rosiglitazone, have been developed to improve insulin sensitivity and stimulate glucose metabolization in muscle and adipose tissues [10]. Paecilocin A, isolated from jellyfish-derived fungus *Paecilomyces variotii*, previously showed good activity in a PPAR-γ binding assay [11]. Subsequently, new phthalimide derivatives were designed as pharmacophores for PPAR-γ activation based on core structures of paecilocin A derivatives and TZDs, including rosiglitazone [14].

In the present study, enhancement of the PPAR-γ agonistic activity of **PD1** was pursued by *in silico* analysis of **PD1**-derived structures, and **PD5** and **PD6** were selected for synthesis and biological evaluation. In contrast to *in silico* predictions, **PD1** consistently exhibited higher PPAR-γ agonistic activity than **PD5** or **PD6** in Ac2F and HepG2 cells. **PD1**–**PD6** were also evaluated with respect to the induction of adipocyte differentiation using murine 3T3-L1 cells, and **PD1** and **PD2** were found to stimulate adipogenesis in a concentration-dependent manner. Furthermore, the adipogenic activity profiles of **PD1**~**PD6** correlated fairly well with their activations of PPAR-γ in Ac2F and HepG2 cells.

In summary, we designed new phthalimide PPAR-γ ligands based on our previous experience and experimental data. The design was aided by SAR analysis and computer simulations of ligand-binding to the LBD of PPAR-γ protein. Synthesized analogues were evaluated for *in vitro* PPAR-γ activation using both murine liver cells (Ac2F) and human liver cells (HepG2). PPAR-γ activation by the compounds was further gauged by induced adipogenesis in 3T3-L1 cells. The presented information on SAR of these phthalimide derivatives would serve as a basis for future study and development of PPAR-γ ligands.
